# Supplementary material for: Patterns of Intron Gain and Loss in Fungi
Source: PLoS Biol. 2004 Nov 30;2(12):e422. doi: 10.1371/journal.pbio.0020422 (PMC532390; doi:10.1371/journal.pbio.0020422)
Supplement: Table S1 — Also available at http://genes.mit.edu/NielsenEtAl/. (4.3 MB ZIP). [file pbio.0020422.st001.zip › NielsenEtAl/html/175.html]

AN5525.1.NCU02366.1.MG03521.1.FG07953.1


```
 CLUSTAL W (1.82) Multiple Sequence Alignments - Introns Inserted


Sequence 1: AN5525.1	766 aa
Sequence 2: FG07953.1	788 aa
Sequence 3: NCU02366.1	784 aa
Sequence 4: MG03521.1	784 aa
Alignment Length: 788 aa
Number Identitical Residues: 539 aa
Alignment Score (without introns) 23822


MG03521.1 	MLALRR~AAAASPLARVSAVTR---RRMATVSDSPLDRK0VRQNNWEE~NNFIPYK~KLS
NCU02366.1	MLASRQ~LLGVARSRAAAGLGR---RCMATATENPLDRK0VKQNLLEE~GNFINYK~KMS
FG07953.1 	MLATRQ~VLGNMSRGRALAGAASGFRRMATVSDNPLDKK0VRQNNWEE~GNYINYK~KMS
AN5525.1  	-----M0LFG--TRG------------MATVAD--LDKK~VEMCNLEK0GNYINYK2KMS
          	         .                 ***.::  **:* *.    *: .*:* ** *:*

MG03521.1 	ENLAIVRSRLNRPLTYAEKILYSHLDNPHEQDIERGVSYLKLRPDRVACQDATAQMAILQ
NCU02366.1	ENLAVVRSRLNRPLTYAEKILYSHLDNPHEQDIERGVSYLRLRPDRVACQDATAQMAILQ
FG07953.1 	ENLDIVRSRLNRPLAYAEKILYSHLADPHGQDIQRGQSYLKLRPDRVACQDATAQMAILQ
AN5525.1  	ENLDVVRRRLTRPLTYAEKILYSHLDDPQNQDIERGKSYLKLRPDRVACQDATAQMAILQ
          	*** :** **.***:********** :*: ***:** ***:*******************

MG03521.1 	FMSAGMPSVANPTTVHCDHLIEAQVGGPKDLERAVNINKEVYDFLASACAKYNIGFWKPG
NCU02366.1	FMSAGMPSVANPTTVHCDHLIEAQVGGEKDLERAININKEVYDFLASACAKYNIGFWKPG
FG07953.1 	FMSAGMDKVANPTTVHCDHLIEAQVGGAKDLARAVDINKEVYNFLSTACAKYDIGFWKPG
AN5525.1  	FMSAGMPSVATPTTVHCDHLIEAQLGGEKDLARANEINKEVYDFLASSTAKYNIGFWKPG
          	****** .**.*************:** *** ** :******:**::: ***:*******

MG03521.1 	SGIIHQILLENYAFPGGLLIGTDSHTPNGGGLGMAAIGVGGADAVDVMAGLPWELKAPKY
NCU02366.1	SGIIHQIILENYAFPGGLLIGTDSHTPNAAGLGMAAIGVGGADAVDVMANLPWELKAPKV
FG07953.1 	SGIIHQILLENYCFPGGLLIGTDSHTVNGGGLGMATIGVGGADAVDVMANLDWELKAPNV
AN5525.1  	SGIIHQIILENYAFPGGLMIGTDSHTPNAGGLAIAAIGVGGADAVDVMAGLPWELKAPKV
          	*******:****.*****:******* *..**.:*:*************.* ******: 

MG03521.1 	IGVHLTGQMSGWTSAKD~IITKLAGITTVKG2GTGHIVEYYG~PGVDTLSSTGA~GTVCN
NCU02366.1	IGVKLTGALNGWTTPKD~VILKLAGILTVKG~GTGSIVEYYG1EGASSMSATGK0ATCGN
FG07953.1 	IGVKLTGQLNGWTAPKD~IILKVADILTVKG~GTGAIVEYHG~PGTDAISCTGM~ATICN
AN5525.1  	IGVRLTGEMSGWTAPKD1IILKVAGLLTVKG~GTGAIIEYHG~PGVNSLSATGM~ATICN
          	***:*** :.***:.** :* *:*.: **** *** *:**:*  *..::*.**  .*  *

MG03521.1 	M~GAEIGATTSVFRFTERMADYLKATKRSDIAEYARAYAKELREDEGAEYDQVIEINLSE
NCU02366.1	M2GAEIGATTSVFPYDKAMFDYLVATKRSDIAEFAKTYQKELQADEGAEYDQHIEINLNE
FG07953.1 	M0GAEIGATTSVFPFNDRMYDYLAATKRKEIGDFARSYAHGLRPDENVEYDQLIEINLSE
AN5525.1  	M~GAEIGATTSLFPFNDRMYDYLKATKRQQIGDFARSYAKDLREDEGAEYDQLIEINLSE
          	* *********:* : . * *** ****.:*.::*::* : *: **..**** *****.*

MG03521.1 	LEPHVNGPFTPDLATPISKFSEAVKANGWPSELKVGLIGSCTNSSYEDMSRGASIARDAL
NCU02366.1	LEPHINGPFTPDLATPISKFAQAAKENNWPEELKVGLIGSCTNSSYEDMTRAASIARDAL
FG07953.1 	LEPHINGPFTPDLGTPISKFSQAVKENGWPEELKVGLIGSCTNSSYEDMTRAASIARDAL
AN5525.1  	LEPHINGPFTPDLATPISQFKEAVKANGWPEELKVGLIGSCTNSSYEDMSRAASIAQDAL
          	****:********.****:* :*.* *.**.******************:*.****:***

MG03521.1 	DHGLKAKTAFTITPGSEQIRATIARDGQLATFEEYGGMVLANACGPCIGQWDRKDVKKGE
NCU02366.1	DHGLKAKAAFVVTPGSEQIRATIARDGQLQTFEEFGGTVLANACGPCIGQWDRRDTPKGT
FG07953.1 	NHGVKAKSAFTVTPGSEQIRATIERDGQLQTFEEFGGIVLANACGPCIGQWDRRDVKKGE
AN5525.1  	DHGLKAKSIFTVTPGSEQIRATIERDGQLKTLEEFGGVILANACGPCIGQWDRKDVKKGT
          	:**:***: *.:*********** ***** *:**:** :**************:*. ** 

MG03521.1 	ANSIVSSYNR~NFTGRNDGNPATHSFVTSPDLVVAMTVAGSLHFNPLTDKLKDKDGNEFM
NCU02366.1	PNSILSSYNR2NFTGRNDGNPATHSFVTSPDLVVAMSIAGSLYFNPLTDSLKDKDGKEFK
FG07953.1 	ANSIISSYNR~NFTGRNDGNPATHSFVASPDLVVAMSIAGSLHFNPLTDTLKDKDGKDFK
AN5525.1  	PNSIVSSYNR~NFTGRNDANPATHAFVTSPDLVVALSIAGTLNFNPLTDTLKDKDGKEFK
          	.***:***** *******.*****:**:*******:::**:* ******.******::* 

MG03521.1 	LKPPTGEALPARGYDAGENTYQAPPKDRASVSVAVNPSSDRLQVLSPFEAWDGKDAKDLP
NCU02366.1	LQAPTGAGLPDRGYDPGQNTYQAPPADRATVQVQVSPTSDRLQVLSPFNAWNGKDATDMP
FG07953.1 	LAPPTGDGLPSRGYDPGQDTYQAPPKDRASVNVDVAPTSDRLQILTPFKPWDGKDAKDMP
AN5525.1  	LKAPTGDGLPSRGYDPGRDTYQAPPTDRSSVDVAVSPSSDRLQLLAGFQPWDGKDATGIP
          	* .*** .** ****.*.:****** **::*.* * *:*****:*: *:.*:****..:*

MG03521.1 	VLIKAQGKTTTDHISMAGPWLKYRGHLDNISNNMLIGAINEANGEANKVKNQLTGEFDAV
NCU02366.1	ILIKAQGKTTTDHISMAGPWLKYRGHLDNISNNMLIGAINAANGEANKVQNFTTGEWDAV
FG07953.1 	ILIKAKGKTTTDHISMAGPWLKYRGHLDNISNNMLIGAINEANDEANKIKNYTTGEWGAV
AN5525.1  	ILIKCQGKTTTDHISMAGPWLKYRGHLDNISNNMLIGAVNAENGEANKIKNVFTGEYGAV
          	:***.:********************************:*  *.****::*  ***:.**

MG03521.1 	PAVARDYKKNGIKWVVIGDWNYGEGSSREHAALEPRHLGGLAIITRSFARI~HETNLKKQ
NCU02366.1	PAVARDYKAKNIPWVVIGDWNYGEGSSREHAALEPRHLGGLAIITRSFARI~HETNLKKQ
FG07953.1 	PAVARDYKSKGIKWVVIGDWNYGEGSSREHAALEPRHLGGLAIITRSFARI~HETNLKKQ
AN5525.1  	PATARDYKARGVKWVVIGDWNYGEGSSREHAALEPRHLGGLAIITRSFARI0HETNLKKQ
          	**.***** ..: ************************************** ********

MG03521.1 	GMLPLTFVDPADYEKIKPDDRVDIKCTELAPGKNITMTVHPKDG-KAFDIPLAHTFNEGQ
NCU02366.1	GMLPLTFADPADYDKISPEDKVDILCTELAVGKPITMRVHPKNG-KDFEVKLNHTFNDAQ
FG07953.1 	GMLPLTFSDPADYDKIRPDDKVDILCTKLQVGKPLPLVVHPADGSKSFEVPLSHTFNEAQ
AN5525.1  	GMLPLTFSDPADYDRIPPDATVDLLCTELAVDKPMTLRVHPKDG-ASFDVKLSHTFNESQ
          	******* *****::* *:  **: **:*  .* :.: *** :*   *:: * ****:.*

MG03521.1 	IEWFKNGSALNTMAKANKN
NCU02366.1	IEWFKNGSALNTMAKNAKN
FG07953.1 	IEWFKNGSALNTMAKATKN
AN5525.1  	IEWFKDGSALNTMARKSGN
          	*****:********:   *
```
